# HDL meets triglyceride

**DOI:** 10.1016/j.jlr.2025.100796

**Published:** 2025-04-04

**Authors:** Tugce Akcan, Fredric B. Kraemer

**Affiliations:** Division of Endocrinology, Gerontology and Metabolism, Stanford University School of Medicine, Stanford, CA, USA

**Keywords:** cardiovascular risk, HDL, triglycerides

## Abstract

The study by *Liu et al* in this issue of the *Journal of Lipid Research* leverages data from the UK Biobank to explore the impact of HDL-TG on atherosclerotic cardiovascular disease risk. The investigators observed that elevated serum triglyceride levels were associated with reduced HDL particle diameter and with increased HDL-TG. Using observational and Mendelian randomization analyses, HDL-TG levels were independently associated with atherosclerotic cardiovascular disease risk even after adjusting for multiple confounders and other risk factors. The results emphasize the need for a broader evaluation of lipid parameters that extends beyond traditional measurements and suggest that incorporating metrics like HDL-TG could enhance risk stratification.

The relationship among HDL-C, plasma triglycerides (TGs), and atherosclerotic cardiovascular disease (ASCVD) has intrigued researchers for decades. Once thought to act as opposing forces in cardiovascular health, HDL-C was historically viewed as protective, while TG were considered secondary risk markers. However, emerging evidence has revealed a more complex connection, challenging these conventional views and leading to a reevaluation of their roles in ASCVD development.

Early population-based studies linked higher HDL-C levels to a reduced risk of ASCVD (1, 2, 3). However, clinical trials designed to raise HDL-C concentrations failed to lower cardiovascular event rates, despite successfully increasing HDL-C levels (4). Observational studies further complicated this understanding by revealing paradoxical findings, very high HDL-C levels were unexpectedly associated with increased ASCVD risk and all-cause mortality (5). Adding to this complexity, Mendelian randomization (MR) studies have shown that genetic polymorphisms influencing HDL-C levels do not necessarily confer cardiovascular protection (6). Conversely, TG, historically regarded as secondary to HDL in cardiovascular risk assessment, have gained recognition as significant contributors to ASCVD risk, with emerging evidence identifying hypertriglyceridemia as a key driver of cardiovascular events (7).

As the understanding of cardiovascular risk drivers evolves, research has increasingly focused on HDL particle composition and functionality, as well as the effects of elevated TG on these properties. Studies have demonstrated that elevated TG levels disrupt HDL particles, notably enriching them with TGs (HDL-TG). This has raised questions about whether HDL-TG could serve as a surrogate marker for cardiovascular risk (8, 9, 10).

In this issue of the *Journal of Lipid Research*, Liu *et al.* present a study leveraging data from the UK Biobank to explore the impact of HDL-TG levels on ASCVD risk. The study found that elevated serum TG levels were associated with adverse alterations in HDL size and composition, including reduced particle diameter and increased HDL-TG, both of which may contribute to elevated ASCVD risk (Figure 1). Using a combination of observational analysis and MR, the authors demonstrated that HDL-TG levels are independently associated with ASCVD risk, even after adjusting for established cardiovascular risk factors and additional confounders, including other lipid parameters. The MR analysis further confirmed HDL-TG as a causal contributor to coronary artery disease (CAD) development, strengthening the validity of these findings. Moreover, the study’s drug-target MR analysis identified omega-3 fatty acids as a promising therapeutic strategy, showing a significant association with lower HDL-TG levels and suggesting a potential avenue for mitigating cardiovascular risk.

**Fig. 1:**
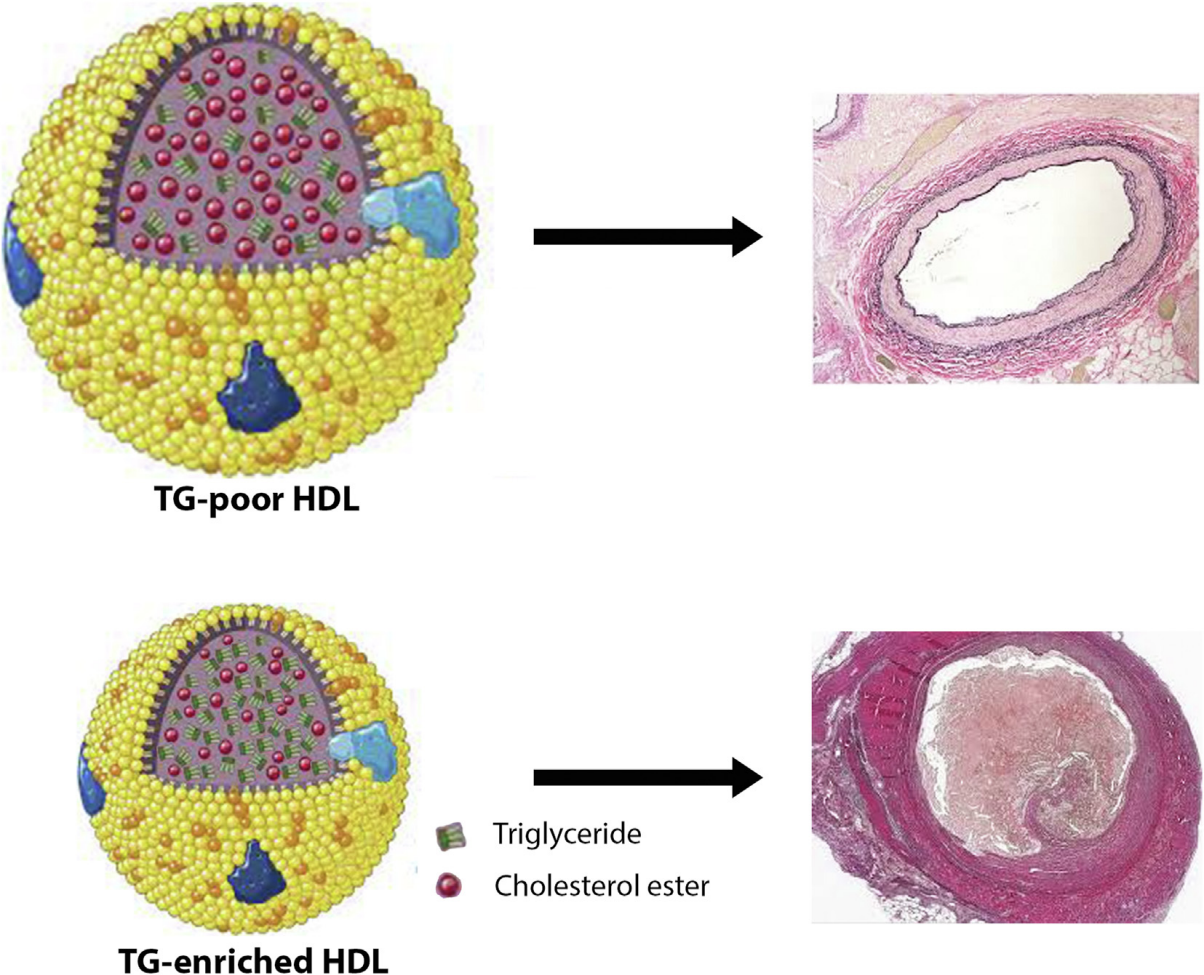
Illustration that triglyceride-rich HDL particles are smaller and have greater predictive value for the development of cardiovascular disease than HDL-cholesterol.

These findings align with previous reports linking elevated TG to HDL dysfunction (8, 9, 10). A small-scale cross-sectional study had previously investigated the relationship between HDL-TG levels and subclinical atherosclerosis in patients with metabolic syndrome and type 2 diabetes (10). However, Liu *et al.*'s study leverages a large prospective cohort and MR analyses, providing stronger evidence for HDL-TG’s role in ASCVD risk.

The mechanisms by which HDL-TG enrichment contributes to CAD development are complex, with Liu *et al.* proposing several pathways. One key mechanism involves cholesteryl ester transfer protein–mediated lipid exchange, in which TG from TG-rich lipoproteins are transferred to HDL in exchange for cholesteryl esters, particularly in the context of elevated TG levels. This exchange generates TG-rich, cholesteryl ester-depleted HDL particles, which can alter the structure of apolipoprotein A-I, HDL's primary protein, potentially impairing its capacity for cholesterol efflux and antioxidant defense. Furthermore, TG-enriched HDL particles may more readily bind to macrophages, promoting cholesteryl ester uptake and encouraging foam cell formation—a critical step in atherosclerosis development (11). Additionally, TG-enriched HDL particles serve as optimal substrates for hepatic lipase, an enzyme crucial to HDL metabolism. Hepatic lipase hydrolyzes HDL-TG, producing smaller, denser HDL particles that are more readily cleared by the liver. This accelerated clearance may further diminish HDL’s protective functions (12).

An important unexplored area in the Liu *et al.* study is the potential alterations of the HDL proteome due to TG enrichment. HDL particles are inherently heterogeneous, comprising a variety of proteins with distinct functional roles (13). Elevated TG levels may cause shifts in the HDL proteome, destabilizing the lipoprotein composition and impairing critical functions such as cholesterol efflux, anti-inflammatory activity, and antioxidant defense. Understanding these complex alterations could provide valuable insights and identify new pathways for ASCVD intervention.

It is noteworthy that this is not the first time that HDL and TG have met in the literature, since more than 20 years ago the TG/HDL-C ratio was suggested to be a surrogate marker for insulin resistance and metabolic dysfunction (14), both major contributors to cardiovascular risk. This raises a critical question: Does the relationship between HDL-TG and ASCVD risk observed by Liu *et al.* represent a distinct pathway contributing to risk, or is it one component of a broader, interconnected process where insulin resistance, metabolic dysfunction, and lipoprotein abnormalities are interrelated aspects of the same underlying condition?

The study by Liu *et al.* offers valuable insights but warrants consideration of certain limitations. While the study adjusted for numerous potential confounders, residual confounding cannot be entirely excluded. Although the analysis accounted for total TG levels, it remains possible that the observed association between HDL-TG levels and CAD risk may be partially mediated by overall TG levels. Additional research is needed to disentangle the independent contributions of total TG and HDL-TG to CAD risk and to better understand their interplay. Given the distinct cardiovascular implications of cholesterol and TG, future studies might benefit from examining the HDL-TG/HDL-C ratio as a potentially informative risk marker. Furthermore, residual pleiotropic effects in the MR analysis could influence the interpretation of causality, necessitating further investigation to validate these findings and clarify causal pathways. Lastly, the metabolic biomarkers in the study were assessed using high-throughput NMR-based lipoprotein profiling via the Nightingale method. While widely utilized, this method has been critiqued compared to alternative approaches for quantifying lipoprotein composition (15).

In summary, ongoing research continues to reveal the complex interplay between HDL, TG, and their roles in the development of CAD. The study by Liu *et al.* highlights the association between HDL-TG and CAD risk, providing important insights into underlying mechanisms and potential biomarkers. These findings emphasize the need for a broader evaluation of lipid profiles that extends beyond traditional measurements. Incorporating metrics like HDL-TG could enhance risk stratification, but a comprehensive approach that considers multiple lipid parameters remains essential for accurately assessing and managing cardiovascular risk.

## Conflict of interest

The authors declare that they have no conflicts of interest with the contents of this article.
